# Consensus recommendations for the diagnosis and management of hemophagocytic lymphohistiocytosis in the Gulf Cooperation Council: a modified Delphi approach

**DOI:** 10.3389/fimmu.2026.1933805

**Published:** 2026-09-14

**Authors:** Mohamed A. Yasin, Muna Al Rasheed, Murtadha Al Khabori, Yasser Wali, Abdelaziz Fahem, Afaf Al Battah, Cigdem Pala Ozturk, Hani Yousif Osman, Maha Sabkar, Mahmoud Marashi, Mozah Al Marshoodi, Nisreen Khalifa, Shehab Mohamed, Salam Al Kindi, Tarek E. Abouzeid, Hind Salama

**Affiliations:** 1 Department of Hematology, Hamad Medical Centre, Doha, Qatar; 2 Department of Internal Medicine and Hematology, Al Kuwait Hospital, Sabah Al-Salem, Kuwait; 3 Department of Hematology, National Hematology and Bone Marrow Transplant Center, University Medical City, Muscat, Oman; 4 Department of Hematology, College of Medicine and Health Sciences, Sultan Qaboos University, Muscat, Oman; 5 Pediatric Hematology/Oncology Unit, Child Health Department, College of Medicine and Health Sciences, Sultan Qaboos University, Muscat, Oman; 6 Hemara Bio, Dubai, United Arab Emirates; 7 Department of Hematology and Bone Marrow Transplant Department, National Centre for Cancer Care and Research, Hamad Medical Corporation, Doha, Qatar; 8 Department of Hematology, Bahrain Oncology Centre, Muharraq, Bahrain; 9 Department of Medicine, Hematology/Oncology Unit, Tawam Hospital, Abu Dhabi, United Arab Emirates; 10 Division of Rheumatology, King Hamad University Hospital, Al Sayh, Bahrain; 11 Department of Hematology, Mediclinic City Hospital, Dubai, United Arab Emirates; 12 Department of Hematology, Sheikh Khalifa Hospital, Fujairah, United Arab Emirates; 13 Pediatric Hematology Oncology Department, National Bank of Kuwait (NBK) Children’s Hospital, Kuwait Ministry of Health, Kuwait City, Kuwait; 14 Department of Hematology, Mouwasat Hospital, Dammam, Saudi Arabia; 15 Department of Adult Hematology, King Abdulaziz Medical City, Ministry of National Guard, Riyadh, Saudi Arabia

**Keywords:** consensus, GCC, hemophagocytic lymphohistiocytosis, HLH, management, treatment

## Abstract

**Background:**

Hemophagocytic lymphohistiocytosis (HLH) is a rare, life-threatening hyperinflammatory syndrome associated with high mortality. Although international recommendations largely guide clinical practice in the Gulf Cooperation Council (GCC) region, the region faces distinct challenges. Therefore, this first GCC-specific consensus study was conducted to develop practical recommendations for the diagnosis and management of HLH in both adult and pediatric patients.

**Methods:**

A modified Delphi methodology was used. A multidisciplinary panel of 15 experts from all GCC countries, comprising 5 steering committee members and 10 extended panel members, was recruited through purposive sampling. A targeted literature review was conducted, and the evidence was graded using the Grading of Recommendations, Assessment, Development and Evaluations (GRADE) framework. Based on the literature review and expert clinical perspectives, 8 clinical domains were selected and 40 gap statements were developed. Following review and revision by the steering committee, the final statements were disseminated to all panel members. Agreement was assessed using a five-point Likert scale, with ≥80% agreement or disagreement prespecified as the threshold for consensus.

**Results:**

All 15 panel members completed the voting process. All 40 statements achieved consensus in round 1, with 80–100% agreement. The recommendations emphasize that HLH assessment should begin as soon as clinical suspicion arises, and that HLH-2004 criteria and HScore should support, rather than replace, clinical judgment. Evaluation should proceed in parallel with investigation for infectious, malignant, rheumatological, and genetic triggers, while treatment should not be delayed in patients with severe or progressive disease. The panel highlighted the importance of early genetic assessment in infants, young children, patients without clear secondary triggers, and families with consanguinity or a suggestive family history. Treatment should be individualized according to age, disease severity, trigger, organ dysfunction, and treatment tolerance, with consideration of targeted and salvage therapies in refractory, relapsed, or treatment-intolerant HLH. Early referral to centers with intensive care, advanced diagnostics, and transplant capability should be prioritized.

**Conclusions:**

These consensus recommendations provide a framework to harmonize HLH recognition, risk-adapted treatment, referral, transplant planning, and regional coordination in GCC countries. Future studies are needed to define regional epidemiology, genetic landscape, treatment responses, and outcomes.

## Introduction

Hemophagocytic lymphohistiocytosis (HLH) is a rare, life-threatening hyperinflammatory syndrome associated with high mortality (1, 2). It is broadly classified into primary and secondary forms. Primary HLH (pHLH), also known as familial HLH (fHLH), is caused by biallelic mutations in genes encoding proteins essential for lymphocyte cytotoxic granule function, including *PRF1*, *UNC13D*, *STXBP2*, and *STX11* (3, 4). Secondary HLH (sHLH), or acquired HLH, is driven by diverse triggers such as infections (most notably Epstein-Barr virus [EBV] and other herpes viruses), hematological malignancies (especially T-cell and NK-cell lymphomas), and autoimmune or autoinflammatory disorders (known as macrophage activation syndrome [MAS]) (1, 5). This classification remains clinically useful; however, the use of the terms “primary” and “secondary” HLH has become increasingly ambiguous with growing recognition of genetic complexity, the role of infections in triggering diverse HLH phenotypes, and variability in how these categories are applied (6).

Without early recognition and aggressive intervention, HLH progresses rapidly to fulminant multisystem organ dysfunction, with reported in-hospital mortality ranging from 20% to over 88% (7). pHLH predominantly affects children and young adults, with an estimated annual incidence of 1.2 per million among children and a mortality rate of 20–30% within the first 2 months after diagnosis (8–10). In contrast, sHLH is more commonly observed in older adults, although reliable prevalence data are currently lacking (8). Despite its rarity, increasing awareness and improved recognition have led to more frequent HLH diagnoses in recent years (11).

Diagnosing HLH remains a major clinical challenge due to the absence of specific laboratory findings or clinical signs (12). It often presents with nonspecific features that mimic more common conditions, including cytopenia, fever, lymphadenopathy, sepsis, hepatosplenomegaly, and multiorgan failure. Consequently, diagnosis is frequently delayed or missed, indicative of a poor prognosis (2, 13, 14). Treatment for HLH is currently anchored in the HLH-94 and HLH-2004 protocols (5, 15). The landmark HLH-2004 cooperative study demonstrated an estimated 5-year survival probability of 61% in children, establishing the etoposide–dexamethasone regimen as the international standard of care (15). In adults, however, these pediatric-derived protocols require individualized modification, given the heterogeneity of underlying triggers, the higher burden of comorbidities, and the recognized risk of treatment-related toxicity from early etoposide use (1, 5). Emerging salvage strategies, including anti-interferon-gamma therapy with emapalumab and checkpoint inhibition for EBV-associated HLH, are broadening therapeutic options, particularly for refractory disease (16, 17). However, allogeneic hematopoietic stem cell transplantation (HSCT) remains the only widely recognized curative approach (15).

Despite these evolving global frameworks, evidence-based guidance tailored to the unique clinical, epidemiological, and genetic background of HLH in the Gulf Cooperation Council (GCC) countries (Bahrain, Kuwait, Oman, Qatar, Saudi Arabia, and UAE) remains limited, potentially differing from Western populations (12, 18). The high rates of consanguinity in several countries across the Middle East and Asia contribute to a higher prevalence of fHLH and a distinct mutational spectrum (19). However, clinical practice in the region is largely guided by international recommendations (1, 3, 20). These recommendations may not fully address local challenges, including differences in genetic background, diagnostic capacity, referral pathways, access to specialized investigations, and treatment availability. These expert-led, consensus-based guidelines aim to harmonize clinical practice for both pediatric and adult patients, address local challenges, and identify priorities for future local studies, regional registries, and collaborative research.

## Methodology

A modified Delphi methodology was used (21), with reporting conducted in accordance with the ACcurate COnsensus Reporting Document (ACCORD) guidelines. This approach combined a comprehensive literature review with expert clinical perspectives (22). The consensus process is outlined in **Figure 1**. Although the study was not prospectively registered, it adhered to established consensus development practices. Formal institutional review board (IRB)/Ethics Committee review was not required as the study did not involve human participants, patient data, or experimental interventions. Participation in the voting process was considered implied consent, granted after participants received relevant study information.

**Figure 1:**
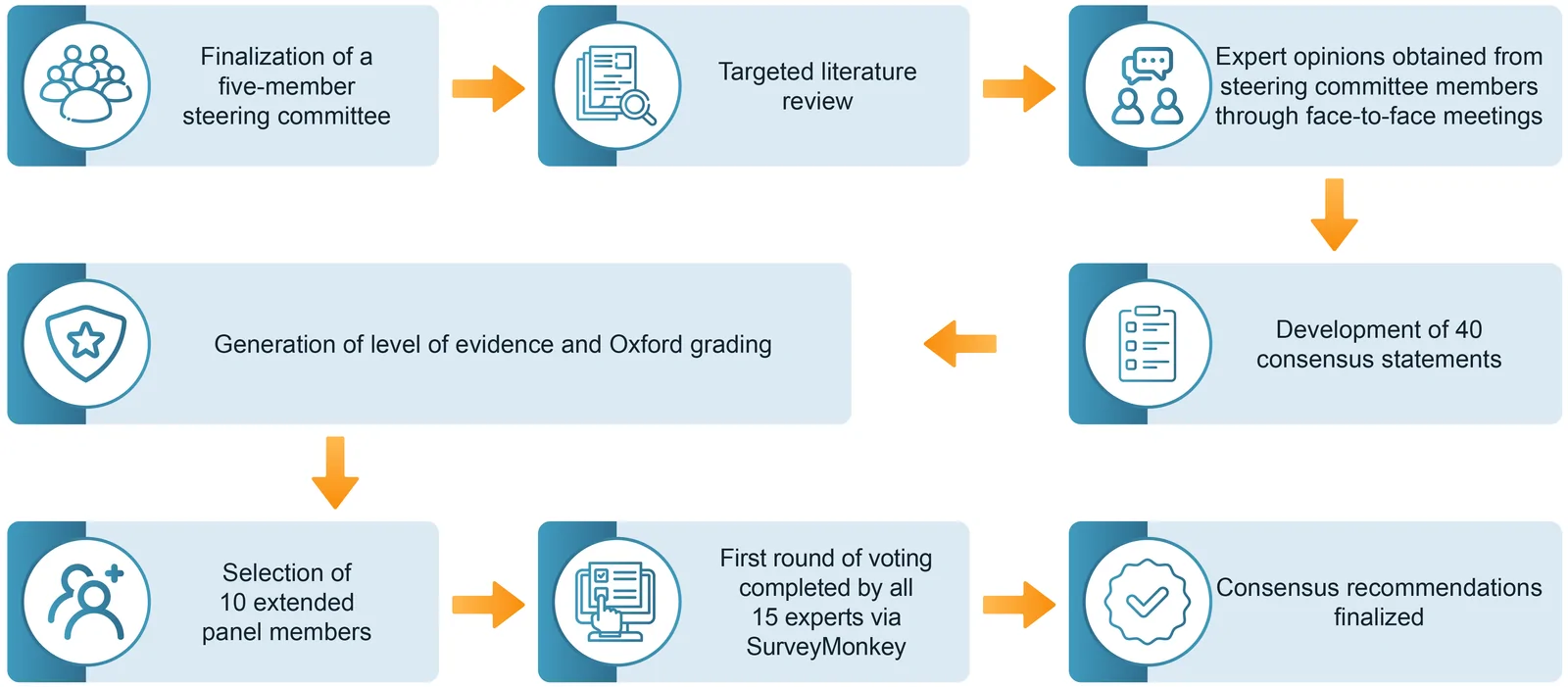
Overview of consensus process.

### Expert panel selection

A steering committee (SC) was established in early December 2025 to define the study objectives. Extended panel members were recruited through purposive sampling, based on their clinical experience, academic contributions, and expertise in HLH management. To ensure balanced representation, experts were drawn from public, academic, and private institutions across GCC. The multidisciplinary panel comprised 15 experts/key opinion leaders, including 5 SC members and 10 extended panel members. The panel included 12 adult hematologists, 2 pediatric hematologists, and 1 rheumatologist. This composition was intentional, as these specialties represent the core disciplines engaged in HLH care across all age groups, ensuring that consensus statements reflect both adult and pediatric clinical perspectives. The panel size is consistent with the recommended range for Delphi studies, ensuring sufficient expertise while facilitating effective discussion and consensus-building (23).

### Literature review

A targeted literature review was conducted to identify gaps and define key areas for consensus. PubMed and Google Scholar were used to identify relevant articles published between January 2012 and May 2026, using a combination of predefined search terms (the full search strategy is provided in **Supplementary Material**). Studies were included if they were original research articles, systematic reviews, meta-analyses, practice guidelines, consensus recommendations, or expert reviews and were published in English. Priority was given to recent publications, international guidelines, and evidence considered clinically relevant to practice in GCC. Preclinical or laboratory-based studies without direct clinical relevance, conference abstracts, case reports, non-English publications, and opinion-based articles without clinical recommendations were excluded. The evidence was then graded according to the Grading of Recommendations, Assessment, Development, and Evaluations (GRADE) criteria (**Supplementary Table 1**) (24, 25). The GRADE assessment was performed by one of the authors, who did not participate in the Delphi voting process. As the assessment was conducted by a single author, no inter-assessor disagreements arose. The reported GRADE categories refer to the certainty/quality of evidence rather than the strength of the recommendations.

### Formulation of the questionnaire

Based on literature review findings and the clinical expertise of the core SC, eight domains were selected for inclusion in the Delphi process (1): early recognition of HLH (2); role of genetics and consanguinity (3); risk stratification and management (4); limitations of conventional therapy (5); refractory, relapsed, or treatment-intolerant HLH (6); special populations (7); referral and transplant; and (8) unmet needs and priorities. The SC’s role was to identify clinical areas with evidence gaps warranting expert consensus and to formulate candidate statements accordingly. The process was designed to be outcome-neutral, with no presumption that consensus would be reached on any individual statement. Across these eight domains, 40 clinically relevant gap statements were developed. These statements, along with supporting evidence, were circulated to all SC members. Remote virtual meetings were then conducted with SC members, and the statements were revised based on their feedback.

### Survey administration and data collection

The finalized questionnaire was disseminated to all 15 panel members via SurveyMonkey. Voting was anonymous, and completion of all statement evaluations was mandatory. Each statement was assessed using a five-point Likert scale: “agree,” “somewhat agree,” “neutral,” “somewhat disagree,” and “disagree.” Panelists who selected “somewhat agree,” “neutral,” “somewhat disagree,” or “disagree” were required to provide a written rationale. Recognizing that certain statements addressed subpopulation-specific clinical scenarios, experts were permitted to indicate if a given statement fell outside their scope of practice. Such responses were recorded as abstentions and excluded from the agreement denominator for that statement.

As there is no universally accepted threshold for defining consensus, consensus levels were defined *a priori* in alignment with the Conducting and REporting of DElphi Studies (CREDES) guidance (26). A threshold of ≥80% agreement or disagreement was prespecified as the criterion for statement acceptance. The process was planned for up to two rounds. Statements not achieving this threshold in round 1 were to be revised based on panelist rationales and resubmitted for a second round of voting. Statements failing to reach the ≥80% threshold after two rounds were to be reviewed by the SC, which would determine, on a case-by-case basis, whether to revise and re-vote, merge with a related statement, or discard. After round 1, the strength of consensus was characterized as high (≥80%) or moderate (55–79%) for descriptive reporting purposes only; this categorization did not alter the acceptance criterion.

Formal pilot testing was not conducted, as the voting statements had been reviewed by the SC before circulation to the wider panel.

## Results

All 15 panel members participated in the voting process. All 40 statements were disseminated for round 1, which commenced on May 27, 2026, and concluded on June 4, 2026, followed by a 1-day analysis. Each statement achieved a high level of consensus in round 1 (80–100% agreement); therefore, a second voting round was not conducted. The final list of consensus statements is provided in **Supplementary Table 2**.

### Early recognition of HLH

Recognizing HLH remains challenging; it often presents as a rapidly progressive hyperinflammatory syndrome with features overlapping sepsis, malignancy, autoimmune disease, MAS, and disseminated infections (27). This overlap is particularly important in acute and critical care settings, where fever, cytopenia, liver dysfunction, coagulopathy, hyperferritinemia, and multiorgan dysfunction may initially be attributed to severe infection or inflammatory shock. Therefore, early recognition of HLH relies less on any single diagnostic marker and more on identifying a pattern of persistent, disproportionate inflammation that does not follow the expected clinical course of the presumed trigger (28).

It is equally important to determine whether the patient has HLH disease or HLH mimic. A patient might fulfil the required number of HLH-2004 criteria or achieve a high HScore; however, severe infections, including disseminated tuberculosis, can reproduce several HLH-like features as part of an appropriate host inflammatory response, thereby risking diagnostic misclassification. In contrast, fHLH and MAS associated with rheumatological disease represent true forms of HLH where uncontrolled immune activation is central to pathogenesis and immunomodulatory treatment is usually required (28). Consequently, the diagnostic process should proceed in parallel: clinicians should assess for HLH early, search for infectious, malignant, rheumatological, and genetic triggers, and exclude mimicking conditions without delaying treatment when clinical suspicion is high.

The HLH-2004 criteria remain the most widely used diagnostic framework. However, in adults, the HLH-probability calculator, or HScore, can offer additional diagnostic support by estimating the probability of reactive HLH using weighted clinical, laboratory, and cytological variables. These include temperature, immunosuppression, organomegaly, cytopenias, triglyceride, ferritin, fibrinogen, aspartate aminotransferase, and hemophagocytosis on bone marrow aspirate (29). Nevertheless, neither HLH-2004 nor the HScore should be used in isolation. Both tools are best interpreted within the broader clinical trajectory, especially when a patient exhibits persistent fever, worsening cytopenias, rising ferritin, evolving liver dysfunction, coagulopathy, neurological features, or progressive organ failure.

The need for early genetic evaluation is especially relevant in the Gulf region. fHLH is classically associated with defects in cytotoxic lymphocyte function and degranulation, most commonly involving *PRF1*, *UNC13D*, *STX11*, and *STXBP2*. Related syndromic forms may involve genes such as *RAB27A* and *LYST* (30). In a Saudi Arabian cohort, 85.7% (48/56) of patients with pHLH had an identifiable genetic mutation. *STXBP2* was the most frequently reported gene, followed by *PRF1*, *RAB27A*, and *UNC13D*, with several variants showing distinct geographic associations (12). The median age at diagnosis was 3 months, and most patients presented with HLH within the first year of life (12). Another case series from Saudi Arabia reported consanguinity in 75% (9/12) of patients and a positive family history in 41%. Nine of the 12 patients were diagnosed with pHLH at a median age of 6 months, further supporting the importance of early molecular testing for suspected HLH in this region (31). A separate study of pediatric patients from Saudi Arabia also showed a predominance of *STXBP2* mutations among patients with fHLH, establishing a genetic diagnosis in 80% of the cohort (32).

In a study of ethnic Omani patients with fHLH, four genetic loci had been described at the time, including FHL2, caused by mutations in *PRF1* and accounting for approximately 20–50% of fHLH cases. Molecular analysis identified seven different mutations in the coding region of the perforin gene, five of which were novel. However, perforin gene defects were not detected in approximately one-third of cases, suggesting additional genetic heterogeneity (33). Overall, data from Oman also indicate that fHLH is relatively prominent in this population, with most cases presenting in infancy. Consanguinity and family history are common, central nervous system (CNS) involvement is frequent, *PRF1* mutations predominate, and delayed diagnosis remains a major challenge (33–36).

In addition to genetic susceptibility, infectious triggers should be considered in the early evaluation of suspected HLH. In regions where visceral leishmaniasis (VL) is endemic, VL should also be considered in children presenting with HLH, as highlighted by a pediatric case report from Oman. This is particularly important because the clinical and biochemical features of VL and HLH may overlap, potentially leading to delayed diagnosis and poorer outcomes (37). Although EBV remains the most frequently reported viral trigger globally (30), data from Saudi Arabia suggest cytomegalovirus (CMV) and other viruses, including rhinovirus, respiratory syncytial virus, herpes simplex virus, and coronavirus, may also serve as important triggers (12). Similarly, in a small Omani cohort, EBV was the most common viral trigger, identified in 47% of cases, followed by CMV in 31%. Viral-associated HLH was more frequently observed in children with a genetic predisposition to HLH or underlying immunodeficiency. A key observation from this cohort was that outcomes were generally favorable in children without a genetic predisposition to HLH, as well as in genetically predisposed children who underwent HSCT (38).

Pediatric cohort data from GCC further highlight the severity of HLH, with many patients requiring critical care and high levels of organ support. These findings reinforce the importance of early diagnosis, prompt initiation of HLH-directed management, and timely consideration of HSCT when indicated (39). Based on international guidelines and GCC data, the expert panel developed the recommendations presented below in **Table 1**.

**Table 1:** Consensus recommendations for early recognition of HLH.

S. No.	Recommendation	Grade of evidence	Level of agreement (%)
1.	All patients with suspected HLH, regardless of age or apparent secondary trigger, should be considered for early comprehensive genetic testing as part of initial evaluation.	Low	80% (12/15)
2.	Given the very early or sepsis-like presentation of HLH in neonates and infants, clinicians should initiate genetic testing together with HLH-directed investigations at first clinical suspicion rather than sequentially after exclusion of secondary causes.	Moderate	93% (13/14)*
3.	The presence of persistent fever (>38.5 °C), cytopenias affecting ≥2 of 3 peripheral blood lineages, and elevated ferritin (≥500 ng/mL in children; HScore ferritin bands of 2,000–6,000 ng/mL or >6,000 ng/mL in adults) should trigger immediate HLH assessment using HLH-2004/2024 in pediatrics and HLH-2004/2024 and/or HScore in adults, together with parallel evaluation for underlying triggers and prompt initiation of algorithm-based HLH management without waiting for confirmatory results.	Moderate	93% (14/15)
4.	In suspected HLH in the GCC, standard workups should include broad viral panels (including HIV, CMV-PCR, EBV-PCR, dengue, influenza, etc.), rather than focusing only on EBV and HIV.	Low	100% (15/15)
5.	Early organ dysfunction in suspected HLH should prompt immediate referral to centers with ICU and HSCT capability to reduce mortality.	High	93% (14/15)

*Abstention was recorded for this statement, as the expert indicated that it was outside their scope of practice.

Level of agreement was calculated as the proportion of respondents selecting either “agree” or “somewhat agree.”

CMV-PCR, cytomegalovirus polymerase chain reaction; EBV-PCR, Epstein-Barr virus polymerase chain reaction; GCC, Gulf Cooperation Council; HIV, human immunodeficiency virus; HLH, hemophagocytic lymphohistiocytosis; HScore, hemophagocytic lymphohistiocytosis probability score; HSCT, hematopoietic stem cell transplantation; ICU, intensive care unit.

### Role of genetics/consanguinity

Current HLH diagnostic criteria do not reliably distinguish patients with significant genetic susceptibility, frequently related to impaired lymphocyte cytotoxic function, from those who develop HLH due to other underlying etiologies (40). A family history, consanguinity, and early age at onset, particularly before 2 years of age, are important indicators that should prompt evaluation for pHLH. Early genetic diagnosis can inform prognosis, guide donor selection for HSCT, and support family counseling (41). The variant profile observed in GCC populations differs substantially from those reported in Western and Asian cohorts (12, 18, 31, 32, 39).

In Saudi Arabia, HLH appears to occur more frequently than in Western populations, potentially due to population structure, consanguinity, and founder effects within specific tribes. In one Saudi Arabian cohort, 91.1% of patients had a family history of consanguinity, and 94.9% were diagnosed with pHLH. Patients were classified as having pHLH based on early disease onset, family history, consanguinity, and/or disease reactivation in the absence of an identifiable trigger, such as infection or autoimmune disease (12). Similarly, data from Qatar demonstrated that 67.8% of patients were diagnosed with pHLH or fHLH, with a documented mortality rate of 42%. Genomic analysis further revealed a population- specific enrichment of potentially deleterious variants in known HLH-related genes. These findings supported previous reports that several rare deleterious variants are more common in Qatar and Oman (18). Such variants may cluster within families or tribes with high levels of inbreeding, highlighting the potential value of region-specific variant panels for screening and early diagnosis. However, access to genetic counseling and genomic services remains limited in parts of the Middle East. Reported barriers include language differences, limited resources, and culturally specific challenges, such as high rates of consanguinity and reluctance to discuss inherited risk openly. Despite these barriers, patients generally demonstrate good coping abilities and an increasingly positive attitude toward genetic testing (42). Notably, in Oman, a specialized genetic counseling clinic for inherited blood disorders, including fHLH, has been established at University Medical City since 2011. The clinic offers counseling and reproductive options, including preimplantation genetic diagnosis, for carrier couples and families with known pHLH (43). These regional experiences highlight both the feasibility and the need to strengthen genetic counseling services and improve access to genomic testing across the region. Based on these data, the expert panel recommends the approaches presented in **Table 2**.

**Table 2:** Consensus recommendations on the role of genetics and consanguinity in HLH.

S. No.	Recommendation	Grade of evidence	Level of agreement (%)
6.	The high prevalence of autosomal recessive pathogenic variants in primary HLH (including *PRF1*, *UNC13D*, *STX11*, and *STXBP2*) supports early genetic evaluation using NGS-based HLH panels, particularly in the setting of parental consanguinity. Where local NGS is unavailable, referral testing should be arranged without delaying management.	Moderate	93% (14/15)
7.	Population-specific enrichment of pathogenic variants in HLH-related genes (such as *PRF1*, *UNC13D*, *RAB27A*, and *LYST*) supports the development of region-specific genetic panels to enhance screening and early diagnosis of primary HLH.	Low	93% (14/15)
8.	Early genetic testing should be strongly considered in GCC patients with HLH without clear secondary triggers, particularly in infants, young children, or consanguineous families, to guide prognosis, HSCT planning, and family counseling.	Moderate	100% (15/15)
9.	Formal integration of genetic counseling into premarital and prenatal care should be recommended to improve carrier screening, reproductive planning, and prevention of inherited genetic disorders.	Moderate	87% (13/15)

Level of agreement was calculated as the proportion of respondents selecting either “agree” or “somewhat agree.”.

GCC, Gulf Cooperation Council; HLH, hemophagocytic lymphohistiocytosis; HSCT, hematopoietic stem cell transplantation; LYST, lysosomal trafficking regulator; NGS, next-generation sequencing; PRF1, perforin 1; RAB27A, RAB27A member RAS oncogene family; STX11, syntaxin 11; STXBP2, syntaxin-binding protein 2; UNC13D, UNC-13 homolog D.

### Risk stratification and management

The main aim of the HLH-94 and HLH-2004 protocols is to induce remission and bridge the patient to HSCT as soon as feasible (30). However, the early introduction of cyclosporine A did not significantly reduce pre-HSCT mortality. Stratifying children with confirmed fHLH by risk group, together with earlier HSCT, may further improve outcomes, with a reported 5-year probability of survival after HSCT of 61% (15, 30). Therefore, treatment may need to be individualized based on patient risk. This could involve less intensive therapy during days 11–42 for selected lower-risk patients, and less reduction of initial therapy during days 43–59 for patients with confirmed fHLH (15). Additionally, surveillance should prioritize the early detection and management of potential complications, including bleeding, respiratory distress, hypotension, neurological involvement, malnutrition, hepatic dysfunction, infection, and other organ failures (39). Evidence-based recommendations are provided in **Table 3**, and the treatment algorithm is presented in **Figure 2**.

**Table 3:** Consensus recommendations on risk stratification and management of HLH.

S. No.	Recommendation	Grade of evidence	Level of agreement (%)
10.	Therapy should be risk-stratified according to disease severity. High-intensity, HLH-directed regimens are indicated for life-threatening hyperinflammation; less intensive approaches are appropriate for milder presentations, balancing therapeutic benefit against treatment-related toxicity.	Moderate	100% (15/15)
11.	Patients with suspected or confirmed HLH should receive immediate supportive care, including hemodynamic stabilization, management of coagulopathy/bleeding, infection coverage, and organ support, as needed.	Moderate	100% (15/15)
Primary/familial HLH			
12.	For primary/familial HLH, HLH-directed therapy, including dexamethasone and etoposide, with or without CNS-directed therapy, should be initiated promptly.	Moderate	100% (15/15)
13.	In patients who respond to induction therapy, treatment should be continued with close monitoring of clinical and laboratory parameters, i.e., fever, ferritin, CBC, fibrinogen, liver function, and CNS status.	Low	100% (15/15)
Secondary HLH			
14.	Concomitant management of the specific underlying trigger alongside HLH-directed therapy improves outcomes and avoids unnecessary immunosuppression when a reversible trigger is present.	Low	100% (15/15)
15.	In severe or progressive disease, treatment should include prompt initiation of corticosteroids and management of the underlying trigger, with consideration of etoposide in severe cases. If etoposide is used, the dose and frequency should be adjusted to 50–100 mg/m² (from 150 mg/m²) and limited to 1–2 doses in older patients.	Moderate	93% (13/14)*
16.	In milder or stable cases, initial management may focus on treating the underlying trigger, with very close monitoring and rapid escalation if there is no clinical response.	Low	93% (14/15)

*Abstention was recorded for this statement, as the expert indicated that it was outside their scope of practice.

Level of agreement was calculated as the proportion of respondents selecting either “agree” or “somewhat agree.”

CBC, complete blood count; CNS, central nervous system; HLH, hemophagocytic lymphohistiocytosis.

**Figure 2:**
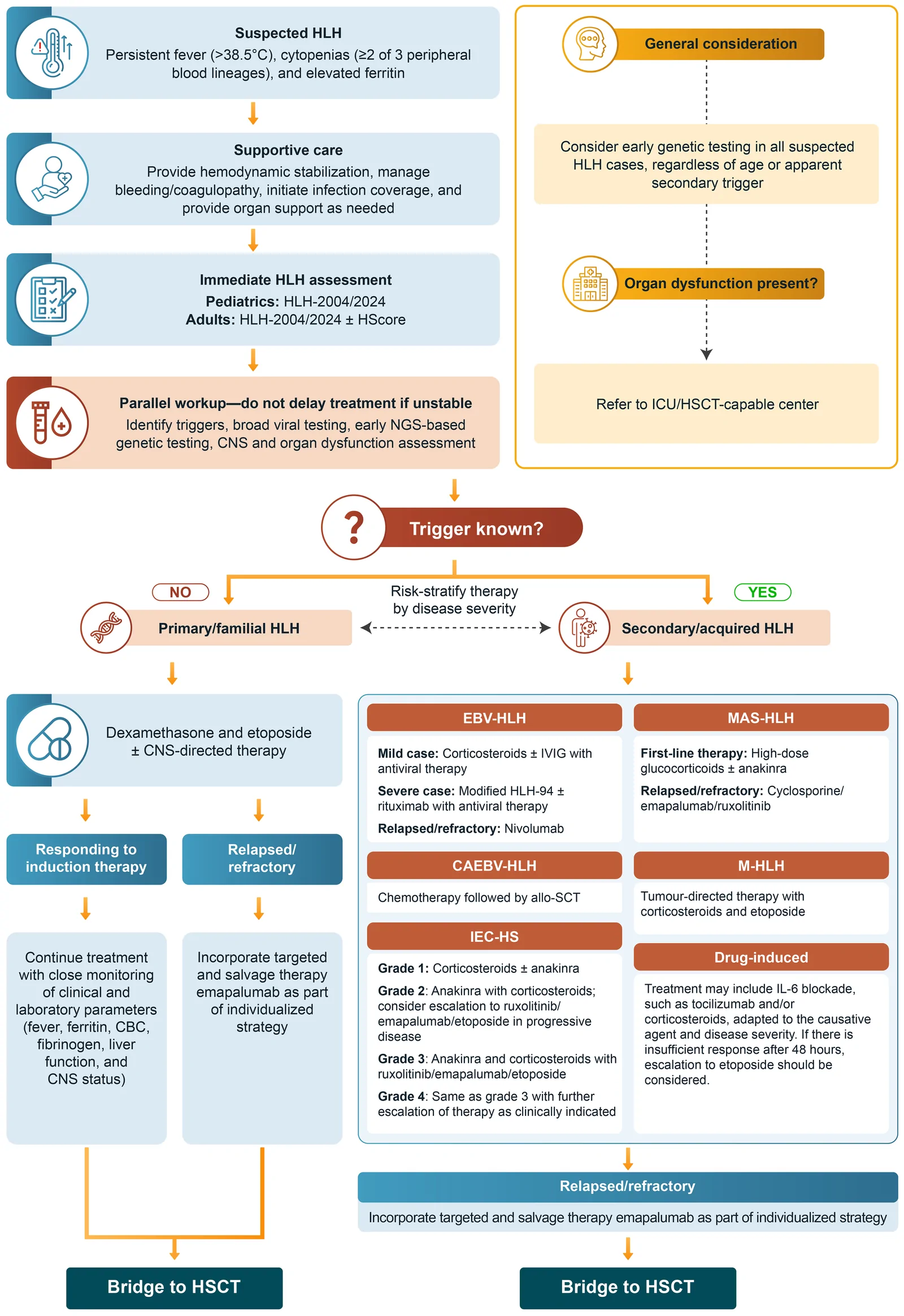
Evidence-based treatment algorithm for HLH. Note: If etoposide is used, the dose and frequency should be adjusted to 50–100 mg/m² (from 150 mg/m²) and limited to 1–2 doses in older patients. Adult HLH treatment cannot be standardized and should be individualized according to the underlying condition and HLH-triggering factors, including infection, malignancy, autoimmune/autoinflammatory disease, drug exposure, or other causes. In secondary HLH, the use of emapalumab for relapsed or refractory disease is off-label but has shown reasonable efficacy. allo-SCT, allogeneic stem cell transplantation; CAEBV-HLH, chronic active Epstein-Barr virus-associated hemophagocytic lymphohistiocytosis; CBC, complete blood count; CNS, central nervous system; EBV-HLH, Epstein-Barr virus-associated hemophagocytic lymphohistiocytosis; HLH, hemophagocytic lymphohistiocytosis; HSCT, hematopoietic stem cell transplantation; ICU, intensive care unit; IEC-HS, immune effector cell–associated hemophagocytic syndrome; IL-6, interleukin-6; IVIG, intravenous immunoglobulin; MAS-HLH, macrophage activation syndrome-associated hemophagocytic lymphohistiocytosis; M-HLH, malignancy-associated hemophagocytic lymphohistiocytosis; NGS, next-generation sequencing. .

### Limitations of conventional therapy

While the HLH-94 and HLH-2004 trials demonstrated the effectiveness of dexamethasone and etoposide as first-line therapy for pHLH, the eligibility criteria used in these studies have since become the basis for diagnosing HLH in routine clinical practice. As a result, meeting these criteria is commonly regarded as sufficient justification for treatment with dexamethasone and etoposide. However, this practice has important limitations, as the criteria are relatively nonspecific and the findings from the HLH-94 and HLH-2004 trials may not be broadly applicable to all patient populations (44).

A key concern with etoposide-based therapy is treatment-related bone marrow suppression, which can increase susceptibility to invasive bacterial or fungal infections in certain patients (45). Additionally, the role of etoposide-based therapy in sHLH remains unclear, particularly in adults, where HLH is almost always secondary. Available data indicate that adult HLH may differ substantially from pediatric disease, with a more aggressive clinical course and poorer survival regardless of treatment (44). Although GCC-specific adult data remain limited, studies from other regions demonstrate poor outcomes with conventional therapy (44). In a single-center retrospective analysis comparing pediatric and adult patients with HLH, the 1-year survival rate was substantially higher among children than adults, at 70% vs. 33%, respectively. Long-term survival remained poor, approximately 29% at 1 year, particularly in adults or those with complex secondary triggers (44, 46). Similarly, a large adult intensive care unit (ICU)-HLH cohort reported 57% mortality, regardless of etiology or specific treatment (47). Regional data from the GCC also suggest substantial mortality: a Saudi Arabia single-center series reported 50% mortality, though this was a small, mixed-age cohort rather than a dedicated adult-only study (31). These findings support the concern that conventional dexamethasone- and etoposide-based therapy may be insufficient to achieve durable disease control in adult HLH, particularly in critically ill patients or those with secondary triggers. This limits the real-world applicability of conventional regimens and increases the need for alternatives with improved safety profiles. Evidence-based recommendations are presented in **Table 4**.

**Table 4:** Limitations of conventional therapy in HLH.

S. No.	Recommendation	Grade of evidence	Level of agreement (%)
17.	Conventional immunochemotherapy (e.g., corticosteroids plus etoposide) is constrained by significant toxicity and limited durable efficacy in adults and complex HLH cases, supporting the need for risk-adapted and alternative therapeutic strategies beyond etoposide-based regimens.	Moderate	100% (15/15)
18.	Standard HLH therapy may be impractical or unsafe in patients with severe organ dysfunction, comorbidities, or frailty, necessitating alternative or tailored approaches.	Moderate	100% (15/15)
19.	As conventional therapy does not directly address persistent triggers, it may be insufficient in cases driven by chronic infection, malignancy, or complex immune dysregulation.	Low	93% (14/15)
20.	There is a need for targeted, risk-adapted therapies to overcome the toxicity, feasibility, and efficacy limitations of conventional treatment in HLH.	Low	100% (15/15)

Level of agreement was calculated as the proportion of respondents selecting either “agree” or “somewhat agree”.

HLH, hemophagocytic lymphohistiocytosis.

### Refractory/relapsed/intolerant HLH

HLH generally results from the combined effects of an exogenous trigger and an underlying genetic predisposition (48). In GCC patients, higher rates of consanguinity may increase the likelihood of genetic predisposition, and persistent triggers can further contribute to refractory or relapsed HLH (12, 31). There is currently no standard treatment for relapsed or refractory HLH. Effective salvage therapies are needed to control disease activity and serve as a bridge to curative HSCT. Emerging agents, including emapalumab, ruxolitinib, nivolumab, and alemtuzumab, have been developed and may represent potential therapeutic options (49).

Emapalumab is the first and only FDA-approved therapy in the United States for pHLH and is indicated for adult and pediatric patients with refractory, recurrent, or progressive disease or intolerance to conventional HLH therapy (50). Its safety and efficacy were evaluated in a phase 2/3 trial in pediatric patients with presumed pHLH. The median time to response was 8 days, with an overall response rate (ORR) of 64.7% among all 34 treated patients (95% CI, 46–80; *P* = 0.0031) and 63% among the 27 patients who had failed prior therapy (95% CI, 42–81; *P* = 0.0134) (51). Additionally, a study evaluating real-world treatment patterns and outcomes in patients treated with ≥1 dose of emapalumab showed a pretransplant survival of 90.5%, with 38 of 42 patients surviving to transplant (16). However, given the limited availability of emapalumab in the GCC, its use may be considered if available; otherwise, treatment should be optimized with other clinically appropriate options such as ruxolitinib. Ruxolitinib has increasingly been explored as salvage therapy for refractory or relapsed HLH. In a trial evaluating ruxolitinib for refractory or relapsed HLH, the ORR was 73.5% (25/34), including a complete response in 14.7% (5/34) and a partial response in 58.8% (20/34). The median time to response was 2 weeks (range, 1–8 weeks) (52). Treatment with emapalumab or ruxolitinib should be individualized based on disease severity, prior therapy, and infection risk (50–52). The following statements provide recommendations for the treatment of refractory/relapsed/intolerant HLH (**Table 5**).

**Table 5:** Consensus recommendations for the treatment of refractory/relapsed/intolerant HLH.

S. No.	Recommendation	Grade of evidence	Level of agreement (%)
21.	In GCC patients, genetic predisposition and persistent triggers substantially increase the likelihood of refractory or relapsed HLH, thereby justifying earlier escalation beyond conventional immunochemotherapy.	Low	100% (15/15)
22.	In GCC patients with refractory, relapsed, or treatment-intolerant HLH, risk-adapted management should incorporate targeted and salvage therapy emapalumab, as part of individualized strategies to achieve disease control and facilitate transition (bridge) to definitive therapies such as HSCT.	Moderate	100% (15/15)

Level of agreement was calculated as the proportion of respondents selecting either “agree” or “somewhat agree.”.

GCC, Gulf Cooperation Council; HLH, hemophagocytic lymphohistiocytosis; HSCT, hematopoietic stem cell transplantation.

### Special populations

Treatment of sHLH focuses on managing the underlying trigger while also controlling hyperinflammation with immunosuppressive therapy, most commonly guided by the HLH-2004 protocol (53). Consensus recommendations for special populations are provided in **Table 6**.

**Table 6:** Consensus recommendations for the treatment of special populations with HLH.

S. No.	Recommendation	Grade of evidence	Level of agreement (%)
23.	In infants and neonates from GCC populations where high consanguinity is associated with an increased burden of pathogenic variants, an HLH-94–based pediatric treatment approach should be considered the consensus mainstay of therapy, with individualized adjustment in neonates according to clinical severity and organ involvement.	Moderate	100% (13/13)*
24.	In MAS-HLH or autoimmune/inflammatory HLH, first-line therapy may include high-dose glucocorticoids with or without early biologic therapy (anakinra). For refractory disease, additional immunomodulatory therapies, including cyclosporine or IFN-γ blockade with emapalumab, may be considered. Ruxolitinib may also be considered in selected refractory MAS-HLH cases, particularly after failure of conventional or etoposide-based therapy.	Moderate	93% (13/14)*
25.	Systematic screening for CNS involvement, coupled with timely and tailored CNS-directed therapy, is of critical importance, particularly in GCC cohorts, where neurological disease occurs with disproportionate frequency and carries significant prognostic impact.	Low	100% (15/15)
26.	Infection-associated HLH, including EBV-HLH and CAEBV-HLH, should be recognized as a phenotype with unique therapeutic needs. In EBV-HLH, corticosteroids with or without IVIG and antiviral therapy may be used in less severe cases, while severe cases may require modified HLH-94; rituximab may be considered to target the EBV B-cell reservoir.	Low	100% (14/14)*
27.	In patients with CAEBV-HLH, chemotherapy followed by allo-SCT may be considered. Nivolumab may also be considered as a potential therapeutic option in selected relapsed or refractory EBV-associated HLH cases.	Low	100% (13/13)*
28.	Malignancy-associated HLH represents a high-risk phenotype that demands integrated cancer and HLH therapy. Corticosteroids may be started promptly, and etoposide may be considered early, including as pre-phase therapy when appropriate.	Low	93% (14/15)
29.	IEC-HS is a distinct HLH phenotype requiring specific diagnostic and management strategies. Treatment should be individualized according to severity grade**: a) Grade 1: Corticosteroids, with or without anakinra, may be considered as first-line therapy, together with intensive supportive care and close monitoring for organ dysfunction. b) Grade 2: Anakinra and corticosteroids may be considered; ruxolitinib, emapalumab, or etoposide may be considered in progressive disease. c) Grade 3: Anakinra and corticosteroids may be considered; ruxolitinib or emapalumab may also be added, with etoposide considered as an alternative. d) Grade 4: Management as for grade 3 may be considered, with further escalation of therapy as clinically indicated.	Low	100% (14/14)*
30.	In drug-induced HLH, the suspected offending agent should be discontinued immediately. Treatment may include IL-6 blockade, such as tocilizumab, and/or corticosteroids, adapted to the causative agent and disease severity. If there is insufficient response after 48 hours, escalation to etoposide may be considered.	Low	100% (14/14)*

*Abstention was recorded for this statement, as the expert indicated that it was outside their scope of practice.

**Grade 1: Asymptomatic or mild symptoms; requires observation and/or clinical and diagnostic evaluations. Intervention is not indicated; Grade 2: Mild to moderate symptoms, with intervention indicated. (e.g., immunosuppressive agents directed at IEC-HS, transfusions for asymptomatic hypofibrinogenemia); Grade 3: Severe or medically significant, but not immediately life-threatening (e.g., coagulopathy with bleeding requiring transfusion support, or hospitalization required for new-onset acute kidney injury, hypotension, or respiratory distress); Grade 4: Life-threatening consequences; urgent intervention indicated (e.g., life-threatening bleeding or hypotension, respiratory distress requiring intubation, dialysis indicated for acute kidney injury)

Level of agreement was calculated as the proportion of respondents selecting either “agree” or “somewhat agree.”

allo-SCT, allogeneic stem cell transplantation; CAEBV, chronic active Epstein-Barr virus; CNS, central nervous system; EBV, Epstein-Barr virus; GCC, Gulf Cooperation Council; HLH, hemophagocytic lymphohistiocytosis; HSCT, hematopoietic stem cell transplantation; IEC-HS, immune effector cell–associated HLH-like syndrome; IFN-γ, interferon gamma; IL, interleukin; IVIG, intravenous immunoglobulin; MAS, macrophage activation syndrome.

### Infants and neonates

The incidence of neonatal HLH is not well established but has been estimated to occur in approximately 1 in 50,000 to 1 in 150,000 live births. The incidence may vary by ethnicity and is higher in populations with increased rates of consanguinity (54, 55). Data on neonatal HLH remain limited, and clinical presentation can be highly variable, ranging from hydrops fetalis, respiratory distress, thrombocytopenia, and sepsis-like illness to multiorgan failure. Herpes simplex virus is among the most commonly reported infectious triggers of neonatal HLH. It should be considered in the differential diagnosis of neonates presenting with sepsis-like illness or unexplained multiorgan dysfunction (54).

In a Saudi pediatric cohort, patients were treated according to either the HLH-94 or HLH-2004 protocol. The median age at fHLH diagnosis was 0.4 years, with 79% of patients being infants. At a median follow-up of 5.6 years, the 5-year overall survival (OS) was 55.4%. Survival was significantly lower in patients with *PRF1* mutations (5-year OS, 21.4%; *P* = 0.008), while HSCT was associated with improved survival (5-year OS, 66.5% vs. 0%; *P* = 0.001) (32). Although HLH-94–based chemoimmunotherapy has substantially improved outcomes in pediatric HLH (56), only a few case reports (57, 58) of neonatal HLH have been published from the GCC region, and dedicated neonatal cohorts are lacking. Collaborative efforts are needed to reduce early mortality, HSCT-related mortality, and long-term neurologic sequelae in this population (56).

### Autoimmune/inflammatory

MAS-HLH is driven by rheumatologic or autoimmune diseases (13). Treatment approaches for MAS remain highly variable, with substantial differences across centers and countries (59). The METAPHOR project was designed to evaluate real-world therapeutic practices for MAS across different clinical settings worldwide through a web-based survey of the pediatric rheumatology community. The survey found that high-dose glucocorticoids, along with interleukin-1 (IL-1) and interferon-gamma (IFN-γ) inhibitors, have demonstrated efficacy in MAS, particularly in systemic juvenile idiopathic arthritis–associated MAS (59).

### Infections

EBV is among the most well-recognized infectious triggers of HLH, often occurring as EBV reactivation during periods of immunosuppression. Less commonly, HLH may be driven by bacterial, parasitic, or fungal infections (13). The prognosis of EBV-HLH has improved substantially with prompt treatment using HLH-94–based protocols; however, the variable severity of EBV-HLH requires individualized adjustment of treatment intensity and duration (1). Combination therapy with intravenous immunoglobulin and corticosteroids appears to be an effective option associated with favorable outcomes in patients with less severe disease (60). Because EBV primarily replicates in B cells, adding rituximab to HLH-directed therapy (at 375 mg/m² weekly for 2–4 doses) may help eliminate the EBV-infected B-cell reservoir in EBV-triggered HLH (61). However, EBV-HLH may relapse or become refractory because etoposide-based regimens do not reliably eradicate the underlying viral reservoir. Nivolumab monotherapy may provide therapeutic benefit by restoring impaired anti-EBV immune responses in this population (17). In addition, nivolumab combined with emapalumab has been reported to induce sustained remission in EBV-associated T/NK-cell lymphoproliferative diseases, potentially reducing the need for HCT in selected patients (62). Further studies are required to confirm its efficacy and define its role in HLH treatment.

Moreover, a high plasma EBV-DNA load, low platelet count, elevated alanine aminotransferase, and cytopenia affecting at least two hematopoietic lineages are associated with an increased risk of progression from chronic active EBV (CAEBV) infection to HLH. A nomogram may be useful for estimating this risk in patients with CAEBV (63). Stem cell transplantation should be considered as a definitive therapeutic option in this case (1).

### Malignancy

Most malignancy-associated HLH (M-HLH) occurs in hematologic cancers, particularly lymphoma and leukemia, but remains underrecognized in adults. Delayed diagnosis may limit HLH-directed therapy, especially when patients present with advanced multiorgan failure. Management should balance control of hyperinflammation with tumor-directed therapy. However, this is challenging because patients with M-HLH are often already myelosuppressed and immunocompromised, making additional cytotoxic therapy with etoposide or anti-thymocyte globulin-based regimens high risk (64). Corticosteroids are commonly used first-line, while dose-adjusted etoposide (50–100 mg/m^2^) may be considered in highly active HLH or impending severe organ damage before initiating malignancy-specific treatment (1, 64).

### Immune effector cell–associated HLH-like syndrome

Immune effector cell–associated HLH-like syndrome (IEC-HS) is a pathological hyperinflammatory syndrome that occurs after immune effector cell therapy, often emerging when cytokine release syndrome (CRS) is resolving or has resolved. It is distinct from CRS and immune effector cell–associated neurotoxicity syndrome and is characterized by features of macrophage activation/HLH, with associated new or worsening cytopenias, hyperferritinemia, coagulopathy with hypofibrinogenemia, and/or transaminitis (65). First-line treatment for IEC-HS generally includes corticosteroids and/or anakinra. Because IEC-HS can be associated with severe and potentially fatal complications, early recognition, comprehensive diagnostic evaluation, and prompt intervention are essential to improve outcomes while preserving the therapeutic benefit of immune effector cell–based therapies. Treatment decisions should be individualized according to patient-specific factors, the nature and severity of toxicities, and available evidence on therapeutic options used in similar clinical scenarios (66, 67).

### Referral and transplant

Although HSCT is the only curative treatment for familial/genetically determined HLH and selected cases of persistent or recurrent HLH, several challenges persist, including refractory hyperinflammation, CNS involvement, limited availability of matched donors, vulnerability to conditioning-related toxicities, and a high incidence of mixed chimerism (68).

In GCC settings, where consanguinity rates are high and founder variants are well recognized, donor identification should be family-based and supported by early human leukocyte antigen (HLA) typing to optimize transplant readiness (12, 18, 31, 32, 68). In addition, experience from Oman suggests that T-cell-depleted haploidentical HSCT may represent a feasible transplant option for patients with HLH in the absence of an HLA-matched related donor (69). Therefore, referral pathways should be standardized to ensure early HSCT consultation.

In the Qatari cohort, where 67.8% of patients were diagnosed with pHLH and fHLH, the disease was confined to the CNS in 26.3%, while overall CNS involvement was identified in 44.4%. Patients with only one diagnostic criterion or those with CNS involvement appeared to benefit most from early molecular workup. This approach enabled timely diagnosis before the disease evolved to its full clinical severity (18). A retrospective/prospective analysis of all patients diagnosed with fHLH in Oman also reported that 56.25% of the patients had evidence of CNS involvement, either at diagnosis or during the course of therapy (35). Therefore, across GCC regions, early referral for HSCT evaluation, including donor search and HLA typing, should be standardized. This may help reduce delays to curative therapy and minimize transplant-related toxicity. The following statements present expert recommendations for HLH referral and transplantation (**Table 7**).

**Table 7:** Consensus recommendations for HLH referral and transplantation.

S. No.	Recommendation	Grade of evidence	Level of agreement (%)
31.	HSCT is indicated for primary/genetic HLH and refractory/relapsed cases, and its early integration should be prioritized in the GCC due to the high prevalence of pathogenic HLH variants.	Moderate	100% (15/15)
32.	Referral timing should prioritize achieving disease remission or minimal active inflammation before HSCT to reduce graft failure and improve outcomes, especially in genetically predisposed populations.	Low	93% (14/15)
33.	Referral pathways should be standardized to ensure early HSCT consultation, donor search, and HLA typing, thereby minimizing delays that compromise transplant success.	Low	100% (15/15)
34.	HSCT conditioning should be individualized, balancing disease eradication and toxicity. Reduced-intensity conditioning is often preferred to reduce early transplant-related morbidity.	Moderate	93% (14/15)
35.	Adults and older patients with HLH should be evaluated for HSCT similarly to pediatric cases in appropriate clinical contexts.	Low	93% (14/15)

Level of agreement was calculated as the proportion of respondents selecting either “agree” or “somewhat agree.”.

GCC, Gulf Cooperation Council; HLA, human leukocyte antigen; HLH, hemophagocytic lymphohistiocytosis; HSCT, hematopoietic stem cell transplantation.

### Unmet needs and priorities

Significant advances have undoubtedly been made in understanding and managing HLH; however, challenges remain. Major unmet needs persist, particularly the lack of prospective clinical trials at a global level. Future studies will require strong collaboration among multiple institutions and investigators, using innovative trial designs capable of generating meaningful results with smaller patient cohorts. Global disparities in access to HLH expertise, specialized diagnostic testing, and novel therapies also present a major challenge and must be addressed through multidisciplinary teams and locally adapted guidelines (70).

Important gaps also persist in both diagnosis and treatment, especially in adults. Most diagnostic criteria are derived from pediatric studies, and the commonly used HLH-2004 criteria have not been validated in adults (1). Therapeutic progress remains limited, with emapalumab currently being the only approved targeted therapy for pHLH. Many treatment approaches still rely on pediatric-based protocols, which may lead to overtreatment and toxicity in adults (1, 45). Experts recommend the evaluation and validation of integrated diagnostic pathways that incorporate structured scoring tools together with selected genetic and functional assays. These pathways could improve diagnostic precision, reduce misdiagnosis, and minimize treatment delays. Additionally, facilitating access to novel agents where possible is crucial. For now, building regional HLH registries across all GCC countries would be an important step forward (**Table 8**).

**Table 8:** Unmet needs and priorities in HLH management.

S. No.	Recommendation	Grade of evidence	Level of agreement (%)
36.	Current HLH diagnostic criteria (including updated HLH-2004) have limitations in specificity for secondary or atypical presentations, and there is a need to evaluate and validate integrated diagnostic pathways, incorporating structured scoring tools and selected genetic/functional assays, to improve diagnostic precision and reduce misdiagnosis and treatment delays.	Moderate	100% (15/15)
37.	Genetic and functional testing should be integrated into diagnostic pathways but must not delay empiric HLH therapy when clinical suspicion is high.	Low	100% (15/15)
38.	For idiopathic HLH, early empiric immunomodulatory treatment should be started in parallel with continued efforts to uncover underlying triggers to improve outcomes.	Low	100% (15/15)
39.	Where access to novel targeted therapies is limited, standardized protocols using available immunomodulatory agents and supportive care should be established, with mechanisms to facilitate access to novel agents where possible.	Low	100% (15/15)
40.	Multidisciplinary care pathways and regional care coordination, supported by diagnostic networks and the establishment of regional registries, are essential to address unmet needs and ensure timely, equitable HLH care. In particular, the current lack of a comprehensive regional registry remains a key gap, limiting visibility of the disease burden, referral patterns, and outcomes across the region.	Low	100% (15/15)

Level of agreement was calculated as the proportion of respondents selecting either “agree” or “somewhat agree.”.

HLH, hemophagocytic lymphohistiocytosis.

## Discussion

This is the first consensus recommendation on HLH for the GCC region. It provides a practical framework to support earlier recognition, risk-adapted treatment, timely referral, and improved regional coordination for both pediatric and adult patients. A key message from this consensus study is that HLH assessment should begin as soon as clinical suspicion arises (1). The panel emphasized that HLH-2004 criteria and HScore are useful supportive tools but should not be interpreted in isolation. This is especially important in adults, where sHLH is more common, triggers are heterogeneous, and the risk of misclassification is high. Therefore, diagnostic evaluation should proceed in parallel with active investigation for infectious, malignant, rheumatological, and genetic causes, while treatment should not be delayed in patients with severe or progressive disease (1, 3).

The GCC setting adds important considerations, including high rates of consanguinity and reported enrichment of pathogenic variants. This supports early genetic evaluation, particularly in infants, young children, patients without clear secondary triggers, and families with consanguinity or suggestive family history (1, 3, 12, 18, 31, 32, 39). However, age alone should not exclude the possibility of an underlying genetic predisposition, and genetic testing may also be considered in older patients with recurrent HLH, atypical or CNS-predominant presentations, familial clustering, or unusually severe HLH triggered by infection. Early molecular diagnosis can clarify prognosis, guide family counseling, support donor selection, and accelerate transplant planning. However, the panel also recognized that genetic testing should complement, not delay, urgent HLH-directed therapy when clinical suspicion is high.

Therapy for HLH needs to be individualized based on disease severity, age, triggers, organ dysfunction, and treatment tolerance. While corticosteroids and etoposides are central to conventional HLH treatment, their toxicity and limited durability in adults and complex sHLH highlight the need for risk-adapted approaches (15, 39). The expert panel also recognizes the emerging role of targeted and salvage therapies, including emapalumab and other immunomodulatory agents, particularly in cases of refractory, relapsed, or treatment-intolerant HLH. Different forms of sHLH also require integrated management that addresses both the hyperinflammatory response and the underlying trigger. This distinction is clinically important, as a uniform treatment approach may result in undertreatment of aggressive HLH or unnecessary toxicity in patients whose disease could be controlled by treating a reversible cause (1, 17, 54, 56, 59, 66). This consensus also highlights the importance of early referral to centers equipped with ICU facilities, advanced diagnostics, and HSCT capabilities. Early donor search, HLA typing, and transplant consultation are particularly relevant in genetically predisposed GCC populations (1, 32, 71). Therefore, the development of standardized referral pathways, multidisciplinary care models, diagnostic networks, and regional registries is essential.

### Strengths and limitations

The rigorous methodology and evidence-based, implementable recommendations are intended to support clinicians in delivering optimal care across the GCC region. The extensive pre-voting review and refinement of the statements by the SC strengthened the clarity and relevance of the consensus statements; however, this process may also have contributed to the high level of agreement achieved in the first Delphi round. Several limitations should also be acknowledged. The Delphi panel was mainly composed of adult hematologists, and the absence of clinical immunologists, geneticists/genetic counselors, infectious disease specialists, and transplant specialists may have limited multidisciplinary input in some consensus areas. In addition, limited prospective data from the GCC region supporting several recommendations necessitated reliance on international evidence and expert opinion. Clinical data on emerging novel agents in GCC populations also remain limited. As frontline HLH treatment continues to evolve rapidly, this guidance should be interpreted alongside newer clinical and real-world evidence published after panel discussions.

## Conclusions

These recommendations provide an important foundation for harmonizing practice while also identifying priorities for future collaborative studies. This represents a practical step toward earlier diagnosis, safer individualized therapy, and more equitable HLH care. Future region-specific studies are necessary to better define the epidemiology, genetic landscape, clinical phenotypes, treatment responses, and long-term outcomes of HLH.

## Data Availability

The original contributions presented int he study are included in the article/supplementary material, further inquiries can be directed to the corresponding author/s.
