# Supplementary material for: Consensus recommendations for the diagnosis and management of hemophagocytic lymphohistiocytosis in the Gulf Cooperation Council: a modified Delphi approach
Source: Front Immunol. 2026 Sep 14;17:1933805. doi: 10.3389/fimmu.2026.1933805 (PMC13616640; doi:10.3389/fimmu.2026.1933805)
Supplement: Supplementary file 1 [file Table1.docx]

**Supplementary Material**

1. **Search strategy:**

("Hemophagocytic Lymphohistiocytosis"[Mesh] OR HLH[tiab] OR "macrophage activation syndrome"[tiab]) AND (diagnos*[tiab] OR "early diagnosis"[tiab] OR HScore[tiab] OR "HLH-2004"[tiab]) AND (genetic*[tiab] OR mutation*[tiab] OR "familial HLH"[tiab] OR "primary HLH"[tiab] OR PRF1[tiab] OR UNC13D[tiab] OR STX11[tiab] OR STXBP2[tiab]) AND (consanguinity[tiab] OR consanguineous[tiab]) AND (prognos*[tiab] OR biomarker*[tiab] OR "risk stratification"[tiab]) AND (etoposide[tiab] OR dexamethasone[tiab] OR cyclosporine[tiab] OR ruxolitinib[tiab] OR emapalumab[tiab] OR alemtuzumab[tiab] OR HSCT[tiab]) AND (refractory[tiab] OR relapsed[tiab] OR "treatment failure"[tiab]) AND (Saudi Arabia[tiab] OR Qatar[tiab] OR Kuwait[tiab] OR UAE[tiab] OR Bahrain[tiab] OR Oman[tiab] OR GCC[tiab])

**Supplementary Table 1. Oxford Grading System**

1A: Level of evidence

| Level of evidence | Therapy/prevention, etiology/harm | Prognosis |
| --- | --- | --- |
| 1a | Systematic review (with homogeneity) of randomized controlled trials (RCTs) | Systematic review (with homogeneity) of inception cohort studies; clinical decision rule validated in different populations |
| 1b | Individual RCT (with narrow confidence interval [CI]) | Individual inception cohort study with >80% follow-up; clinical decision rule validated in a single population |
| 1c | All or none | All or none case series |
| 2a | Systematic review (with homogeneity) of cohort studies | Systematic review (with homogeneity) of either retrospective cohort studies or untreated control groups in RCTs |
| 2b | Individual cohort study (including low**-**quality RCTs; e.g. <80% follow-up) | Retrospective cohort study or follow-up of untreated control patients in an RCT; derivation of a clinical decision rule or validated on a split-sample only |
| 2c | "Outcomes" research and ecological studies | "Outcomes" research |
| 3a | Systematic review (with homogeneity) of case–control studies |  |
| 3b | Individual case–control study |  |
| 4 | Case series (and poor-quality cohort and case–control studies) | Case series (and poor-quality prognostic cohort studies) |
| 5 | Expert opinion without explicit critical appraisal, or based on physiology, bench research, or "first principles" | Expert opinion without explicit critical appraisal, or based on physiology, bench research, or "first principles" |

1B: Quality of evidence

| Code | Quality of evidence | Definition |
| --- | --- | --- |
| A | High | Further research is very unlikely to change our confidence in the estimate of effect.   - Several high‐quality studies with consistent results - In special cases: one large, high‐quality multicenter trial |
| B | Moderate | Further research is likely to have an important impact on our confidence in the estimate of effect and may change the estimate.   - One high‐quality study - Several studies with some limitations |
| C | Low | Further research is very likely to have an important impact on our confidence in the estimate of effect and is likely to change the estimate.   - One or more studies with severe limitations |
| D | Very low | Any estimate of the effect is very uncertain.   - Expert opinion - No direct research evidence - One or more studies with very severe limitations |

**Supplementary Table 2. Consensus recommendations with level and grade of evidence**

| Statement | Evidence | Study type | Level of evidence | Grade of evidence | Level of agreement (%) |  |  |  |  |
| --- | --- | --- | --- | --- | --- | --- | --- | --- | --- |
| Section 1: Early Recognition of HLH | | | | |  |  |  |  |  |
| 1. All patients with suspected HLH, regardless of age or apparent secondary trigger, should be considered for early comprehensive genetic testing as part of initial evaluation. | Presentations and outcomes of familial hemophagocytic lymphohistiocytosis in the pediatric intensive care units (PICUs) (Alsohime et al. 2023)  Genetic and clinical characteristics of pediatric patients with familial hemophagocytic lymphohistiocytosis (Ahmari et al. 2021)  Genetic background of primary and familial HLH in Qatar: Registry data and population study (Elgaali et al. 2024)  Hemophagocytic lymphohistiocytosis: Single-center series of 12 cases from Saudi Arabia (Elyamany et al. 2016)  The 2022 EULAR/ACR points to consider at the early  stages of diagnosis and management of suspected  haemophagocytic lymphohistiocytosis/macrophage  activation syndrome (HLH/MAS) (Shakoory et al. 2023) | Retrospective cohort study  Retrospective cohort study  Retrospective cohort study  Retrospective case-series  Consensus guidelines | 4  4  4  4  5 | Low | 80% (12/15) |  |  |  |  |
| 2. Given the very early or sepsis-like presentation of HLH in neonates and infants, clinicians should initiate genetic testing together with HLH-directed investigations at first clinical suspicion rather than sequentially after exclusion of secondary causes. | Presentations and outcomes of familial hemophagocytic lymphohistiocytosis in the pediatric intensive care units (PICUs) (Alsohime et al. 2023)  Neonatal hemophagocytic lymphohistiocytosis: A meta-analysis of 205 cases (Kranz et al. 2024) | Multicenter retrospective cohort study  SLR of observational studies | 3b  2a | Moderate | 93% (13/14)* |  |  |  |  |
| 3. The presence of persistent fever (>38.5°C), cytopenias affecting ≥2 of 3 peripheral blood lineages, and elevated ferritin (≥500 ng/mL in children; HScore ferritin bands 2,000–6,000 or >6,000 ng/mL in adults) should trigger immediate HLH assessment using HLH-2004/2024 in pediatrics and HLH-2004/2024 and/or Hscore in adults, together with parallel evaluation for underlying triggers and prompt initiation of algorithm-based HLH management without waiting for confirmatory results. | Hemophagocytic lymphohistiocytosis in critically ill patients: Diagnostic reliability of HLH-2004 criteria and HScore (Knaak et al. 2020)  Recommendations for the management of hemophagocytic lymphohistiocytosis in adults (La Rosée et al. 2019) | Validation studies  Consensus recommendations | 2a  5 | Moderate | 93% (14/15) |  |  |  |  |
| 4. In suspected HLH in the GCC, standard workups should include broad viral panels (including HIV, CMV-PCR, EBV-PCR, dengue, influenza etc.), rather than focusing only on EBV and HIV. | Outcome of viral-associated hemophagocytic lymphohistiocytosis at a tertiary hospital (Nazir et al. 2022)  Recommendations for the management of hemophagocytic lymphohistiocytosis in adults (La Rosée et al. 2019)  Hemophagocytic lymphohistiocytosis: Single-center series of 12 cases from Saudi Arabia (Elyamany et al. 2016) | Retrospective study  Consensus recommendations  Retrospective case-series | 3b  5  4 | Low | 100% (15/15) |  |  |  |  |
| 5. Early organ dysfunction in suspected HLH should prompt immediate referral to centers with ICU and HSCT capability to reduce mortality. | Treatment and mortality of hemophagocytic lymphohistiocytosis in critically ill children: A systematic review and meta-analysis (Tan et al. 2023)  The 2022 EULAR/ACR points to consider at the early  stages of diagnosis and management of suspected  haemophagocytic lymphohistiocytosis/macrophage  activation syndrome (HLH/MAS) (Shakoory et al. 2023) | Meta-analysis  Consensus guidelines | 1a  5 | High | 93% (14/15) |  |  |  |  |
| Role of Genetics/Consanguinity |  |  |  |  |  |  |  |  |  |
| 6. The high prevalence of autosomal recessive pathogenic variants in primary HLH (including *PRF1, UNC13D, STX11, and STXBP2*) supports early genetic evaluation using NGS-based HLH panels, particularly in the setting of parental consanguinity. Where local NGS is unavailable, referral testing should be arranged without delaying management. | Genetic and clinical characteristics of pediatric patients with familial hemophagocytic lymphohistiocytosis (Ahmari et al. 2021)  Genetic background of primary and familial HLH in Qatar: Registry data and population study (Elgaali et al. 2024)  Diagnostic guidelines for familial hemophagocytic lymphohistiocytosis revisited (Henter et al. 2024) | Retrospective cohort study   Retrospective cohort study  Expert consensus | 3b  3b  5 | Moderate | 93% (14/15) |  |  |  |  |
| 7. Population‑specific enrichment of pathogenic variants in HLH‑related genes (such as *PRF1, UNC13D, RAB27A, LYST*) supports development of region‑specific genetic panels to enhance screening and early diagnosis of primary HLH. | Genetic and clinical characteristics of pediatric patients with familial hemophagocytic lymphohistiocytosis (Ahmari et al. 2021)  Genetic background of primary and familial HLH in Qatar: Registry data and population study (Elgaali et al. 2024)  Hemophagocytic lymphohistiocytosis: Single-center series of 12 cases from Saudi Arabia (Elyamany et al. 2016)  Clinical presentations, diagnosis, and genetic features of hemophagocytic lymphohistiocytosis: A single institutional experience with the Saudi population (Alradhi et al. 2024) | Retrospective cohort study  Retrospective cohort study  Retrospective case-series  Retrospective cohort study | 3b  3b  4  3b | Low | 93% (14/15) |  |  |  |  |
| 8. Early genetic testing should be strongly considered in GCC patients with HLH without clear secondary triggers, particularly in infants, young children, or consanguineous families, to guide prognosis, HSCT planning, and family counseling. | Presentations and outcomes of familial hemophagocytic lymphohistiocytosis in the pediatric intensive care units (PICUs) (Alsohime et al. 2023)  Genetic and clinical characteristics of pediatric patients with familial hemophagocytic lymphohistiocytosis (Ahmari et al. 2021)  Genetic background of primary and familial HLH in Qatar: Registry data and population study (Elgaali et al. 2024)  Diagnostic guidelines for familial hemophagocytic lymphohistiocytosis revisited (Henter et al. 2024) | Multicenter retrospective cohort study  Retrospective cohort study  Retrospective cohort study  Expert consensus | 3b  3b  3b  5 | Moderate | 100% (15/15) |  |  |  |  |
| 9. Formal integration of genetic counseling into premarital and prenatal care should be recommended to improve carrier screening, reproductive planning, and prevention of inherited genetic disorders. | Premarital screening and genetic counseling program: Studies from an endogamous population (Bener, Al-Mulla, and Clarke 2019)  Awareness of premarital screening and genetic counseling among Saudis and its association with sociodemographic factors: A national study (Al-Shroby et al. 2021)  Qatar’s genetic counseling landscape: Current insights and future prospects (Abiib et al. 2024) | Cross-sectional observational study  Cross-sectional observational study  Review | 3b  3b  5 | Moderate | 87% (13/15) |  |  |  |  |
| Section 3: Risk Stratification and Management | | | | |  |  |  |  |  |
| 10. Therapy should be risk-stratified according to disease severity. High-intensity, HLH-directed regimens are indicated for life-threatening hyperinflammation; less intensive approaches are appropriate for milder presentations, balancing therapeutic benefit against treatment-related toxicity. | Confirmed efficacy of etoposide and dexamethasone in HLH treatment: Long-term results of the cooperative HLH-2004 study (Bergsten et al. 2017)  Presentations and outcomes of familial hemophagocytic lymphohistiocytosis in the pediatric intensive care units (PICUs) (Alsohime et al. 2023) | Prospective cohort study  Retrospective cohort study | 2b  3b | Moderate | 100% (15/15) |  |  |  |  |
| 11. Patients with suspected or confirmed HLH should receive immediate supportive care, including hemodynamic stabilization, management of coagulopathy/bleeding, infection coverage, and organ support as needed. | Presentations and outcomes of familial hemophagocytic lymphohistiocytosis in the pediatric intensive care units (PICUs) (Alsohime et al. 2023)  Recommendations for the management of hemophagocytic lymphohistiocytosis in adults (La Rosée et al. 2019)  Intensive care management of hemophagocytic lymphohistiocytosis: A case series (Traversi et al. 2025)  Coagulation disorders in patients with severe hemophagocytic lymphohistiocytosis (Valade et al. 2021)  Management of complex infections in hemophagocytic lymphohistiocytosis in adults (Zhang et al. 2023) | Multicenter retrospective cohort study  Consensus recommendations  Case-series  Prospective study  Review | 3b  5  4  2b  5 | Moderate | 100% (15/15) |  |  |  |  |
| *Primary/familial HLH* |  |  |  |  |  |  |  |  |  |
| 12. For primary/familial HLH, HLH-directed therapy should be initiated promptly, including dexamethasone and etoposide with or without CNS-directed therapy. | Chemoimmunotherapy for hemophagocytic lymphohistiocytosis: Long-term results of the HLH-94 treatment protocol (Trottestam et al. 2011)  Confirmed efficacy of etoposide and dexamethasone in HLH treatment: Long-term results of the cooperative HLH-2004 study (Bergsten et al. 2017) | Prospective cohort study  Prospective cohort study | 2b  2b | Moderate | 100% (15/15) |  |  |  |  |
| 13. In patients who respond to induction therapy, treatment should be continued with close monitoring of clinical and laboratory parameters, i.e. fever, ferritin, CBC, fibrinogen, liver function, and CNS status. | Serum ferritin is a good indicator for predicting the efficacy of adult HLH induction therapy (Hua et al. 2022)  Recommendations for the management of hemophagocytic lymphohistiocytosis in adults (La Rosée et al. 2019) | Retrospective study  Consensus recommendations | 3b  5 | Low | 100% (15/15) |  |  |  |  |
| *Secondary HLH* |  |  |  |  |  |  |  |  |  |
| 14. Concomitant management of the specific underlying trigger alongside HLH-directed therapy improves outcomes and avoids unnecessary immunosuppression when a reversible trigger is present. | Recommendations for the management of hemophagocytic lymphohistiocytosis in adults (La Rosée et al. 2019)  A retrospective review of secondary hemophagocytic lymphohistiocytosis (HLH) and dengue-associated HLH from a teaching hospital in Singapore (Tso et al. 2024) | Consensus recommendations  Retrospective study | 5  3b | Low | 100% (15/15) |  |  |  |  |
| 15. In severe or progressive disease, treatment should include prompt initiation of corticosteroids and management of the underlying trigger, with consideration of etoposide in severe cases. If etoposide is used, the dose and frequency should be adjusted to 50–100 mg/m² (from 150 mg/m²) and limited to 1–2 doses for older patients. | Confirmed efficacy of etoposide and dexamethasone in HLH treatment: Long-term results of the cooperative HLH-2004 study (Bergsten et al. 2017)  Recommendations for the management of hemophagocytic lymphohistiocytosis in adults (La Rosée et al. 2019) | Prospective cohort study  Consensus recommendations | 2b  5 | Moderate | 93% (13/14)* |  |  |  |  |
| 16. In milder or stable cases, initial management may focus on treating the underlying trigger, with very close monitoring and rapid escalation if there is no clinical response. | Recommendations for the management of hemophagocytic lymphohistiocytosis in adults (La Rosée et al. 2019) | Consensus recommendations | 5 | Low | 93% (14/15) |  |  |  |  |
| Section 4: Limitations of Conventional Therapy | | | | | |  |  |  |  |
| 17. Conventional immunochemotherapy (e.g., corticosteroids plus etoposide) is constrained by significant toxicity and limited durable efficacy in adults and complex HLH cases, supporting the need for risk‑adapted and alternative therapeutic strategies beyond etoposide‑based regimens. | The efficacy of etoposide-based therapy in adult secondary hemophagocytic lymphohistiocytosis (Naymagon, Tremblay, and Mascarenhas 2021)  Improved survival outcomes with anakinra over etoposide-based therapies for the management of adults with hemophagocytic lymphohistiocytosis: A retrospective multicenter research network study (Lee 2024)  Perspectives on the current diagnostic and treatment paradigms in secondary hemophagocytic lymphohistiocytosis (HLH) (Naymagon et al. 2025) | Retrospective comparative cohort study  Retrospective comparative cohort study  Review | 2b  2b  5 | Moderate | 100% (15/15) |  |  |  |  |
| 18. Standard HLH therapy may be impractical or unsafe in patients with severe organ dysfunction, comorbidities, or frailty, necessitating alternative or tailored approaches. | Prognostic factors and outcomes in adults with secondary hemophagocytic lymphohistiocytosis: A single-center experience (Apodaca et al. 2018)  Prognostic factors and outcomes of adults with hemophagocytic lymphohistiocytosis (Parikh et al. 2014) | Prospective study  Comparative study | 2b  2b | Moderate | 100% (15/15) |  |  |  |  |
| 19. As conventional therapy does not directly address persistent triggers, it may be insufficient in cases driven by chronic infection, malignancy, or complex immune dysregulation. | Perspectives on the current diagnostic and treatment paradigms in secondary hemophagocytic lymphohistiocytosis (HLH) (Naymagon et al. 2025)  Recommendations for the management of hemophagocytic lymphohistiocytosis in adults (La Rosée et al. 2019) | Review  Consensus recommendations | 5  5 | Low | 93% (14/15) |  |  |  |  |
| 20. There is a need for targeted, risk‑adapted therapies to overcome the toxicity, feasibility, and efficacy limitations of conventional treatment in HLH. | Perspectives on the current diagnostic and treatment paradigms in secondary hemophagocytic lymphohistiocytosis (HLH) (Naymagon et al. 2025)  Recommendations for the management of hemophagocytic lymphohistiocytosis in adults (La Rosée et al. 2019) | Review  Consensus recommendations | 5  5 | Low | 100% (15/15) |  |  |  |  |
| Section 5: Refractory/Relapsed/Intolerant HLH | | | | |  |  |  |  |  |
| 21. In GCC patients, genetic predisposition and persistent triggers substantially increase the likelihood of refractory or relapsed HLH, thereby justifying earlier escalation beyond conventional immunochemotherapy. | Genetic background of primary and familial HLH in Qatar: Registry data and population study (Elgaali et al. 2024)  Hemophagocytic lymphohistiocytosis: Single-center series of 12 cases from Saudi Arabia (Elyamany et al. 2016) | Retrospective cohort study  Case series | 3b  4 | Low | 100% (15/15) |  |  |  |  |
| 22. In GCC patients with refractory, relapsed, or treatment-intolerant HLH, risk-adapted management should incorporate targeted and salvage therapy with emapalumab as part of individualized strategies to achieve disease control and facilitate transition (bridge) to definitive therapies such as HSCT. | Emapalumab in children with primary hemophagocytic lymphohistiocytosis (Locatelli et al. 2020)  Real-world treatment patterns and outcomes in patients with primary hemophagocytic lymphohistiocytosis treated with emapalumab (Chandrakasan et al. 2024)  Clinical management of relapsed/refractory hemophagocytic lymphohistiocytosis in adult patients: A review of current strategies and emerging therapies (Yildiz et al. 2021) | Phase 2-3 trial  Retrospective cohort study  Review | 2b  3b  5 | Moderate | 100% (15/15) |  |  |  |  |
| Section 6: Special Populations | | | | |  |  |  |  |  |
| 23. In infants and neonates from GCC populations where high consanguinity is associated with an increased burden of pathogenic variants, an HLH-94–based pediatric treatment approach should be considered the consensus mainstay of therapy, with individualized adjustment in neonates according to clinical severity and organ involvement. | Chemoimmunotherapy for hemophagocytic lymphohistiocytosis: Long-term results of the HLH-94 treatment protocol (Trottestam et al. 2011)  Genetic and clinical characteristics of pediatric patients with familial hemophagocytic lymphohistiocytosis (Ahmari et al. 2021) | Prospective cohort study  Retrospective cohort study | 2b  3b | Moderate | 100% (13/13)* |  |  |  |  |
| 24. In MAS-HLH or autoimmune/inflammatory HLH, first-line therapy may include high-dose glucocorticoids with or without early biologic therapy (anakinra). For refractory disease, additional immunomodulatory therapies, including cyclosporine or IFN-γ blockade with emapalumab, may be considered. Ruxolitinib may also be considered in selected refractory MAS-HLH cases, particularly after failure of conventional or etoposide-based therapy. | Current treatment in macrophage activation syndrome worldwide: A systematic literature review to inform the METAPHOR project (Baldo et al. 2025)  Evidence-based diagnosis and treatment of macrophage activation syndrome in systemic juvenile idiopathic arthritis (Boom et al. 2015) | SLR of observational studies  Review | 2a  5 | Moderate | 93% (13/14)* |  |  |  |  |
| 25. Systematic screening for CNS involvement, coupled with timely and tailored CNS-directed therapy, is of critical importance, particularly in GCC cohorts, where neurologic disease occurs with disproportionate frequency and carries significant prognostic impact. | Genetic background of primary and familial HLH in Qatar: Registry data and population study (Elgaali et al. 2024)  Presentations and outcomes of familial hemophagocytic lymphohistiocytosis in the pediatric intensive care units (PICUs) (Alsohime et al. 2023) | Retrospective cohort study  Retrospective cohort study | 3b  3b | Low | 100% (15/15) |  |  |  |  |
| 26. Infection‑associated HLH, including EBV‑HLH and CAEBV‑HLH, should be recognized as phenotypes with unique therapeutic needs. In EBV-HLH, corticosteroids with or without IVIG and antiviral therapy may be used in less severe cases, while severe cases may require modified HLH-94; rituximab may be considered to target the EBV B-cell reservoir. | Efficient management of secondary haemophagocytic lymphohistiocytosis with intravenous steroids and γ-immunoglobulin infusions (Georgiadou et al. 2019)  Requirement for etoposide in the initial treatment of Epstein-Barr virus-associated haemophagocytic lymphohistiocytosis (Song, Wang, and Wang 2019)  Recommendations for the management of hemophagocytic lymphohistiocytosis in adults (La Rosée et al. 2019)  Treatment of Epstein-Barr virus-induced haemophagocytic lymphohistiocytosis with rituximab-containing chemo-immunotherapeutic regimens (Chellapandian et al. 2013)  Development of a nomogram to predict the risk of chronic active Epstein-Barr virus infection progressing to hemophagocytic lymphohistiocytosis (He, Wang, Song, et al. 2022)  Rituximab-containing immuno-chemotherapy regimens are effective for the elimination of EBV for EBV-HLH with only and mainly B lymphocytes of EBV infection (Meng et al. 2021) | Retrospective cohort study  Retrospective cohort study  Consensus recommendations  Retrospective case series  Retrospective cohort study  Retrospective case series | 3b  3b  5  4  3b  4 | Low | 100% (14/14)* |  |  |  |  |
| 27. In patients with CAEBV-HLH, chemotherapy followed by allo-SCT may be considered. Nivolumab may also be considered as a potential therapeutic option in selected relapsed or refractory EBV-associated HLH cases. | Recommendations for the management of hemophagocytic lymphohistiocytosis in adults (La Rosée et al. 2019)  Nivolumab treatment of relapsed/refractory Epstein-Barr virus-associated hemophagocytic lymphohistiocytosis in adults (Liu et al. 2020)  Development of a nomogram to predict the risk of chronic active Epstein-Barr virus infection progressing to hemophagocytic lymphohistiocytosis (He, Wang, Song, et al. 2022) | Consensus recommendations  Retrospective case series  Retrospective cohort study | 5  4  3b | Low | 100% (13/13)* |  |  |  |  |
| 28. Malignancy‑associated HLH represents a high‑risk phenotype that demands integrated cancer and HLH therapy. Corticosteroids may be started promptly, and etoposide may be considered early, including as pre-phase therapy when appropriate. | Recommendations for the management of hemophagocytic lymphohistiocytosis in adults (La Rosée et al. 2019) | Consensus recommendations | 5 | Low | 93% (14/15) |  |  |  |  |
| 29. IEC-HS is a distinct HLH phenotype requiring specific diagnostic and management strategies. Treatment should be individualized according to severity grade** (a) Grade 1: Corticosteroids, with or without anakinra, may be considered as first-line therapy, together with intensive supportive care and close monitoring for organ dysfunction. (b) Grade 2: Anakinra and corticosteroids may be considered; ruxolitinib, emapalumab, or etoposide may be considered in progressive disease. (c) Grade 3: Anakinra and corticosteroids may be considered; ruxolitinib or emapalumab may also be added, with etoposide considered as an alternative. (d) Grade 4: Management as for Grade 3 may be considered, with further escalation of therapy as clinically indicated. | Immune effector cell-associated hemophagocytic lymphohistiocytosis-like syndrome (IEC-HS) (Hines et al. 2023)  How I treat HLH-like toxicities after immune effector cell therapy (Johnson et al. 2026) | Consensus recommendations  Retrospective case series | 5  4 | Low | 100% (14/14)* |  |  |  |  |
| 30. In drug-induced HLH, the suspected offending agent should be discontinued immediately. Treatment may include IL-6 blockade such as tocilizumab and/or corticosteroids, adapted to the causative agent and disease severity. If there is insufficient response after 48 hours, escalation to etoposide may be considered. | Recommendations for the management of hemophagocytic lymphohistiocytosis in adults (La Rosée et al. 2019) | Consensus recommendations | 5 | Low | 100% (14/14)* |  |  |  |  |
| Section 7: Referral and Transplant | | | | | |  |  |  |  |
| 31. HSCT is indicated for primary/genetic HLH and refractory/relapsed cases, and its early integration should be prioritized in the GCC due to high prevalence of pathogenic HLH variants. | Stem cell transplantation for children with hemophagocytic lymphohistiocytosis: Results from the HLH-2004 study (Bergsten et al. 2020)  Genetic and clinical characteristics of pediatric patients with familial hemophagocytic lymphohistiocytosis (Ahmari et al. 2021)  Primary hemophagocytic lymphohistiocytosis: A severe immune dysregulatory disease with various genotypic features and outcomes: A cross-sectional study from a tertiary pediatric center (Öztürk et al. 2026)  Outcomes of allogeneic hematopoietic stem cell transplantation for primary hemophagocytic lymphohistiocytosis in adults (He et al. 2022)  Recommendations for the management of hemophagocytic lymphohistiocytosis in adults (La Rosée et al. 2019) | Prospective study  Retrospective cohort study  Cross-sectional study  Retrospective observational study  Consensus recommendations | 2b  3b  4  4  5 | Moderate | 100% (15/15) |  |  |  |  |
| 32. Referral timing should prioritize achieving disease remission or minimal active inflammation before HSCT to reduce graft failure and improve outcomes, especially in genetically predisposed populations. | Primary hemophagocytic lymphohistiocytosis: a severe immune dysregulatory disease with various genotypic features and outcomes: a cross-sectional study from a tertiary pediatric center (Öztürk et al. 2026) | Cross-sectional study | 4 | Low | 93% (14/15) |  |  |  |  |
| 33. Referral pathways should be standardized to ensure early HSCT consultation, donor search, and HLA typing, thereby minimizing delays that compromise transplant success. | Primary hemophagocytic lymphohistiocytosis: a severe immune dysregulatory disease with various genotypic features and outcomes: a cross-sectional study from a tertiary pediatric center (Öztürk et al. 2026)  Recommendations for the management of hemophagocytic lymphohistiocytosis in adults (La Rosée et al. 2019) | Cross-sectional study  Consensus recommendations | 4  5 | Low | 100% (15/15) |  |  |  |  |
| 34. HSCT conditioning should be individualized, balancing disease eradication and toxicity. Reduced intensity conditioning is often preferred to reduce early transplant‑related morbidity. | Reduced-intensity conditioning for hematopoietic cell transplant for HLH and primary immune deficiencies (Allen et al. 2018) | Prospective phase 2 trial | 2a | Moderate | 93% (14/15) |  |  |  |  |
| 35. Adults and older patients with HLH should be evaluated for HSCT similarly to pediatric cases in appropriate clinical contexts. | Recommendations for the management of hemophagocytic lymphohistiocytosis in adults (La Rosée et al. 2019) | Consensus recommendations | 5 | Low | 93% (14/15) |  |  |  |  |
| Section 8: Unmet Needs and Priorities | | | | | |  |  |  |  |
| 36. Current HLH diagnostic criteria (including updated HLH-2004) have limitations in specificity for secondary or atypical presentations, and there is a need to evaluate and validate integrated diagnostic pathways, incorporating structured scoring tools and selected genetic/functional assays, to improve diagnostic precision and reduce misdiagnosis and treatment delays. | Validation of the HScore and the HLH-2004 diagnostic criteria for the diagnosis of hemophagocytic lymphohistiocytosis in a multicenter cohort (Bilston et al. 2022)  Performances of the H-Score for diagnosis of hemophagocytic lymphohistiocytosis in adult and pediatric patients (Debaugnies et al. 2016) | Validation study  Validation study | 2a  2a | Moderate | 100% (15/15) |  |  |  |  |
| 37. Genetic and functional testing should be integrated into diagnostic pathways but must not delay empiric HLH therapy when clinical suspicion is high. | Diagnostic guidelines for familial hemophagocytic lymphohistiocytosis revisited (Henter et al. 2024)  Consensus recommendations for the diagnosis and management of hemophagocytic lymphohistiocytosis associated with malignancies (Lehmberg et al. 2015) | Consensus recommendations  Consensus recommendations | 5  5 | Low | 100% (15/15) |  |  |  |  |
| 38. For idiopathic HLH, early empiric immunomodulatory treatment should be started in parallel with continued efforts to uncover underlying triggers to improve outcomes. | Recommendations for the management of hemophagocytic lymphohistiocytosis in adults (La Rosée et al. 2019) | Consensus recommendations | 5 | Low | 100% (15/15) |  |  |  |  |
| 39. Where access to novel targeted therapies is limited, standardized protocols using available immunomodulatory agents and supportive care should be established, with mechanisms to facilitate access to novel agents where possible. | Recommendations for the management of hemophagocytic lymphohistiocytosis in adults (La Rosée et al. 2019) | Consensus recommendations | 5 | Low | 100% (15/15) |  |  |  |  |
| 40. Multidisciplinary care pathways and regional care coordination, supported by diagnostic networks and the establishment of regional registries, are essential to address unmet needs and ensure timely, equitable HLH care. In particular, the current lack of a comprehensive regional registry remains a key gap, limiting visibility of the disease burden, referral patterns, and outcomes across the region. | Recommendations for the management of hemophagocytic lymphohistiocytosis in adults (La Rosée et al. 2019) | Consensus recommendations | 5 | Low | 100% (15/15) |  |  |  |  |

*Abstention was recorded for this statement, as the expert indicated that it was outside their scope of practice.

**Grade 1: Asymptomatic or mild symptoms; requires observation and/or clinical and diagnostic evaluations. Intervention not indicated; Grade 2: Mild to moderate symptoms, with intervention indicated. (e.g., immunosuppressive agents directed at IEC-HS, transfusions for asymptomatic hypofibrinogenemia); Grade 3: Severe or medically significant, but not immediately life-threatening (e.g., coagulopathy with bleeding requiring transfusion support, or hospitalization required for new-onset acute kidney injury, hypotension or respiratory distress); Grade 4: Life-threatening consequences: urgent intervention indicated (e.g., life-threatening bleeding or hypotension, respiratory distress requiring intubation, dialysis indicated for acute kidney injury).

**Note:** Level of agreement was calculated as the proportion of respondents selecting either "agree" or "somewhat agree."
